# 1,6-Naphthyridin-2(1*H*)-ones: Synthesis and Biomedical Applications

**DOI:** 10.3390/ph14101029

**Published:** 2021-10-09

**Authors:** Juan Marcos Oliveras, Raimon Puig de la Bellacasa, Roger Estrada-Tejedor, Jordi Teixidó, José I. Borrell

**Affiliations:** Grup de Química Farmacèutica, IQS School of Engineering, Universitat Ramon Llull, Via Augusta 390, E-08017 Barcelona, Spain; juanoliverasj@iqs-blanquerna.url.edu (J.M.O.); raimon.puig@iqs.url.edu (R.P.d.l.B.); roger.estrada@iqs.url.edu (R.E.-T.); jordi.teixido@iqs.edu (J.T.)

**Keywords:** 1,6-naphthyridin-2(1*H*)-one, substitution pattern, synthesis, biological activity

## Abstract

Naphthyridines, also known as diazanaphthalenes, are a group of heterocyclic compounds that include six isomeric bicyclic systems containing two pyridine rings. 1,6-Naphthyridines are one of the members of such a family capable of providing ligands for several receptors in the body. Among such structures, 1,6-naphthyridin-2(1*H*)-ones (**7**) are a subfamily that includes more than 17,000 compounds (with a single or double bond between C3 and C4) included in more than 1000 references (most of them patents). This review will cover the analysis of the diversity of the substituents present at positions N1, C3, C4, C5, C7, and C8 of 1,6-naphthyridin-2(1*H*)-ones, the synthetic methods used for their synthesis (both starting from a preformed pyridine or pyridone ring), and the biomedical applications of such compounds.

## 1. Introduction

At the beginning of any research project aimed at the development of new potential drug candidates for the treatment of a certain disease, one of the most important decisions to be taken is the selection of the central molecular structure (*scaffold*) on which to introduce the substituents needed to interact with the corresponding biological receptor. Such *scaffolds* can be selected based on the natural ligands of the receptor, the synthetic background of the research group, or, frequently, using the so-called *privileged heterocyclic structures*, a concept introduced by Evans in the late 1980s [1,2].

Such privileged structures are usually heterocyclic compounds such as quinoline, benzimidazole, pyrazole, indole, piperazine, and others, that are present in many drugs developed throughout the history of medicinal chemistry. Another example of such privileged heterocycles are pyrido[2,3-*d*]pyrimidine structures and, more particularly, pyrido[2,3-*d*]pyrimidin-7(8*H*)-ones [3] that have allowed our group to describe compounds with nM activities as breakpoint-cluster-region protein (BCR) kinase inhibitors for B lymphoid malignancies [4], discoidin domain-containing receptor 2 (DDR2) inhibitors for treatment of lung cancer [5], such as hepatitis C virus (HCV) inhibitors [6], and other biological activities.

**Figure 1:**
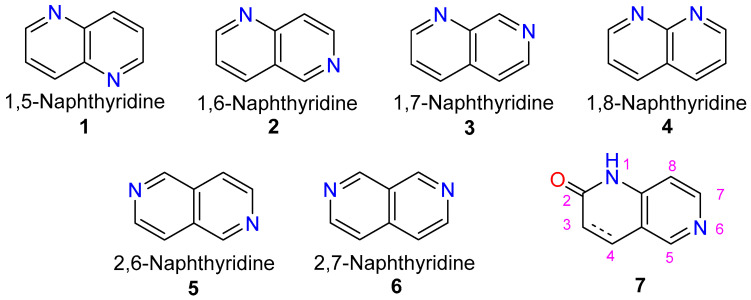
Structures of the isomeric naphthyridines (**1**–**6**) and 1,6-naphthyridin-2(1*H*)-ones (**7**).

Similar structures are naphthyridines, also called pyridopyridines and benzodiacins, a group of diazanaphthalene compounds composed of six isomeric heterocyclic systems containing two pyridine rings. They can be divided into two small groups: the 1,X-naphthyridines (X = 5, 6, 7, 8) and the 2,X-naphthyridines (X = 6, 7) (Figure 1) [7]. Since the synthesis by Reissert in 1893 of the first naphthyridine, who proposed the given name, we had to wait until 1927 when the unsubstituted 1,5-naphthyridine (**1**) and 1, 8 naphthyridine (**4**) were synthesized. Finally, the family was completed with the synthesis in 1958 of 1,6-(**2**), 1,7-(**3**), and 2,7-naphthyridine (**6**), and the isolation of 2,6-naphthyridine (**5**) in 1965.

**Scheme 1:**
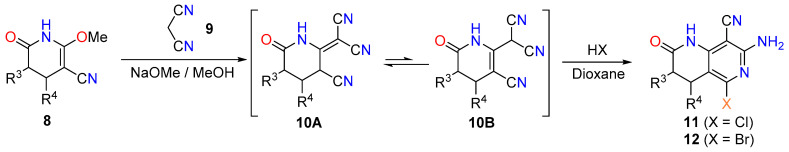
Synthesis of 7-amino-5-halo substituted 1,6-naphthyridin-2(1*H*)-ones (**11**, X = Cl or **12**, X = Br) from 2-methoxy-6-oxo-1,4,5,6-tetrahydropyridine-3-carbonitriles (**8**).

Among this group of isomeric naphthyridines, the 1,6-naphthyridin-2(1*H*)-ones (**7**) (Figure 1) were especially relevant for our group because in the late 1990s we developed a series of synthetic protocols for such structures from 2-methoxy-6-oxo-1,4,5,6-tetrahydropyridine-3-carbonitriles (**8**)—the same starting product we used for pyrido[2,3-*d*]pyrimidin-7(8*H*)-ones [8,9]. Thus, for instance, the treatment of **8** with malononitrile (**9**) in NaOMe/MeOH afforded the tautomeric system **10A** ↔ **10B**, in which the equilibrium is shifted towards the **10A** form. The subsequent treatment with hydrogen halide in dioxane always yields the corresponding 7-amino-5-halo system (**11**, X = Cl or **12**, X = Br) regardless of the hydrogen halide employed (HCl or HBr) and the thermal level used (Scheme 1).

Our conviction that structures **7** could be excellent *scaffolds* for the development of potential drug candidates impelled us to carry out a preliminary search in SciFinder [10], revealing that there are more than 1000 references, including over 17,000 compounds, showing both C3-C4 single and double bonds. Over the past 10 years, the number of references including 1,6-naphthyridin-2(1*H*)-ones substructures have increased exponentially, evincing the interest in these systems and their potential biomedical applications. In fact, most of the reported reviews involving these scaffolds (28 in total) are focused on their biological activity, as will be seen later, with the last one covering structural and synthetic aspects of such structures from 2005 [11]. Consequently, the lack of a specific revision of 1,6-naphthyridin-2(1*H*)-ones (**7**) impelled us to review the literature covering structural, synthetic, and biological aspects.

## 2. Structural Features of 1,6-Naphthyridin-2(1*H*)-ones: Substitution Patterns and Degree of Unsaturation C3-C4

An important aspect to be considered when a certain *scaffold* is selected to start a project looking for new biological activities and patents is to have an idea of the diversity already covered in the Markush formula based on such a structure. It is worth noting that this information was virtually inaccessible until the emergence of computerized databases, such as SciFinder.

**Figure 2:**
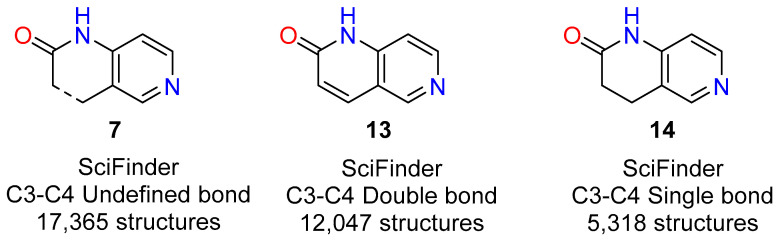
Number of 1,6-naphthyridin-2(1*H*)-ones retrieved using an *undefined bond* (**7**), double bond (**13**) and single bond (**14**) between C3 and C4, respectively.

To analyze the particular case of 1,6-naphthyridin-2(1*H*)-ones (**7**), we determined the total number of structures **7** included in the database. By means of the *Unspecified bond* tool in the substructural search, single and double bonds were concurrently considered between C3 and C4 (depicted by a discontinuous line in **7**, Figure 2). The search gave a total number of 17,365 1,6-naphthyridin-2(1*H*)-ones (**7**), a number that is slowly but continuously growing (the latest search was performed in January 2021). Then, we carried out searches with a C3-C4 double bond **13** (12,047 structures) and a C3-C4 single bond **14** (7067 structures) (Figure 2). It has already been described [3] that a single-bond search may include some double-bonded structures. Therefore, the resulting search was curated using the *Substract* command of the *Combine Answer Sets* tool in SciFinder to obtain the final set of 5318 molecules with a C3-C4 single bond (Figure 2). It is interesting to note that from the total number of structures **7** included in SciFinder, 70% correspond to structures presenting a C3-C4 double bond. Such a ratio in favor of the C3-C4 double bond could be due both to structural requirements for the biological activity of compounds 7 (mostly used as tyrosine kinase inhibitors as described later) or to the easier synthetic approaches for such unsaturated structures.

Structures **13**, which present a C3-C4 double bond, are included in around 970 references. The interest in such structures is shown by around 450 patents (46.4%). In contrast, around 120 references (a number clearly lower than the preceding one) include structures **14** with a C3-C4 single bond, but remarkably 50% are patents.

Given that the maximum number of structures that can be downloaded from SciFinder as an SDFile is limited to 500, performing a diversity analysis with specialized software is not possible. Consequently, we decided to explore one-by-one the substitution patterns at positions C3, C4, C5, C7, C8, and N1 for each degree of C3-C4 unsaturation in order to have a picture of the diversity covered by the substances already described.

### 2.1. Substitution Pattern at N1

The analysis of the substitution pattern at N1 of the 1,6-naphthyridin-2(1*H*)-ones with a C5-C6 single bond (**14**) and with a C5-C6 double bond (**13**) (Table 1) shows that compounds **14** have been usually left unsubstituted at N1 (R^1^ = H, 51.86% of structures described), while compounds **13** are usually substituted at such a position (almost 64% of structures), with R^1^ = Me and R^1^ = Ph as the most used substituents. Such differences seem to relate to the different biological activities these two families of structures are oriented towards. In Table 1 (and the following tables), the different substitution patterns are illustrated with representative references.

### 2.2. Substitution Pattern at C3 and C4

In this family of compounds, the substitution pattern at C3 and C4 is usually connected with the relative selectivity between biological receptors. As will be described later in the biological section, 1,6-naphthyridin-2(1*H*)-ones (**14**), with a C3-C4 single bond, and 1,6-naphthyridin-2(1*H*)-ones (**13**), bearing a C3-C4 double bond, present very different substitution patterns at C3 and C4 and, correspondingly, have been addressed to very different biological targets.

Thus, in the case of the structures **14** (C3-C4 single bond), 32.37% present at least a substituent at C3 and a CH_2_ at C4 [8,34], while only 0.85% present a substituent at C4 and a CH_2_ at C3 [9,35]. Only 1.22% present one substituent both at C3 and C4 [36,37], and 3.74% of the structures do not present substituents at C3 nor at C4 [38,39]. These substitution patterns cover 38.18% of the total diversity, with the rest covered by more complex substitution patterns.

On the contrary, in the case of the structures **13** (C3-C4 double bond), 33.80% present only a substituent at C3 (R^4^ = H), with a phenyl ring in almost half of them. In only 0.75% of the structures is there a substituent at C4 (R^3^ = H), while in 28.51% of the structures R^3^ = R^4^ = H. In this case, such substitution patterns cover 63.06% of the total diversity.

These results clearly show that the substitution pattern at C3 and C4 of the 1,6-naphthyridin-2(1*H*)-ones with a C3-C4 single bond (**14**) is quite rich both in the level of substitution on each carbon atom and on the nature of the substituents present (although almost one-third of the compounds described present a single substituent at C3). In comparison, in the case of the 1,6-naphthyridin-2(1*H*)-ones bearing a C3-C4 double bond (**13**), the most common situations are with a substituent at C3 or with no substituents at positions C3 and C4.

### 2.3. Substitution Pattern at C5 and C7

The analysis of the diversity at positions C5 and C7 of naphthyridines **14** and **13**, bearing a C3-C4 single and double bond, respectively, has focused on the following types of substituents: H, C (either alkyl groups or aromatic rings), N (primary amines, aminoalkyl, or aminoaryl groups or heterocyclic rings connected by the nitrogen atom), O (hydroxy group probably as the carbonyl tautomer, ethers, or ester groups), and halogen. The results obtained are included in Table 2 and Table 3, respectively.

In the case of 1,6-naphthyridin-2(1*H*)-ones (**14**) with a C3-C4 single bond (Table 2), the most commonly found situation is the absence of any substituent at position C5 (R^5^ = H), which covers almost 79% of the available diversity. The presence of carbon substituents is quite low (around 1%), while nitrogen substituents are even lower (0.77%). Only oxygen and halogen substituents are present in relevant amounts (around 5% and 15%, respectively). The diversity considered covers the 99.97% diversity at the C5 position. As for position C7, once more the most abundant situation (65.87%) is the absence of any substituent (R^7^ = H), followed by the presence of a carbon substituent (25.65%). Both situations cover roughly 92% of the substitution patterns at C7 in structures **14**.

On the other hand, in 1,6-naphthyridin-2(1*H*)-ones (**13**) bearing a C3-C4 double bond (Table 3), once more the most common situation is the absence of any substituent at C5 (R^5^ = H), which covers around 67% of the diversity. In second position, we found carbon substituents (around 21%), followed by oxygen, and nitrogen substituents (8.25% and 3.92%, respectively). Such substitution patterns cover almost 99% of the diversity at such positions. As for position C7, the carbon substituents cover 43.25% of the diversity, which added to the compounds not presenting a substituent at such position (R^7^ = H, 33.98%) with those presenting a nitrogen substituent (16.34%) cover most of the diversity at such positions (almost 94%). The combination R^5^ = H and R^7^ = alkyl group covers 46% of all compounds (see for instance Shao [45]).

### 2.4. Substitution Pattern at C8

A total of 4927 (92.65%) 1,6-naphthyridin-2(1*H*)-ones (**14**) with a C3-C4 single bond bear no substituent at position C8 (R^8^ = H) [58,59], and only 7.03% have a carbon substituent [20,31,60,61,62]. Particularly interesting is the protocol developed by our group that allows the presence of a nitrile group at such positions that can be further derivatized [8,9]. In the case of 1,6-naphthyridin-2(1*H*)-ones (**13**) with a C3-C4 double bond, the percentage of unsubstituted structures at position C8 is reduced to 78.52% (9459 structures) [19,63], while the presence of carbon substituents rises to 20.15% (2427 compounds) [29,31,32,56,64,65,66,67,68,69].

**Figure 3:**
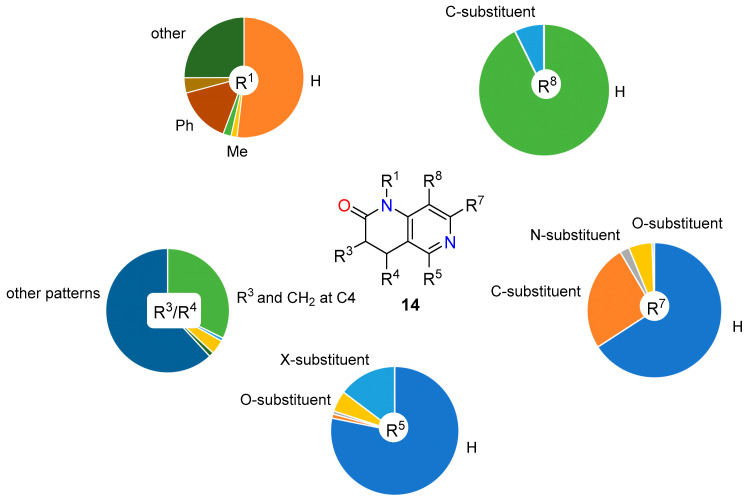
Diversity analysis of the substituents present at positions C3, C4, C5, C7, C8, and N1 of 1,6-naphthyridin-2(1*H*)-ones (**14**) with a C3-C4 single bond included in Scifinder.

**Figure 4:**
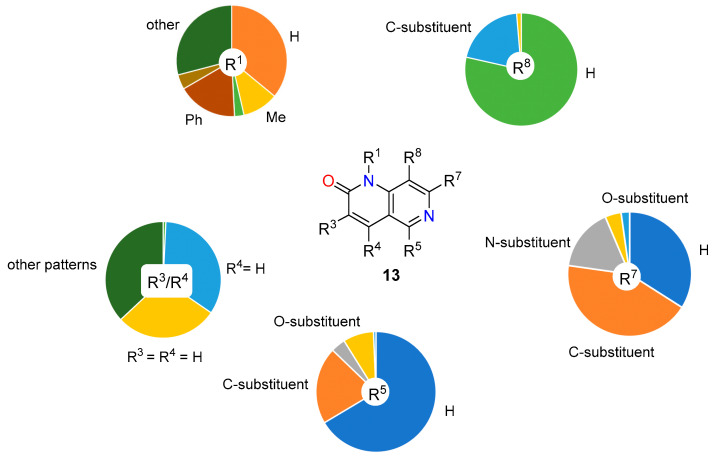
Diversity analysis of the substituents present at positions C3, C4, C5, C7, C8, and N1 of 1,6-naphthyridin-2(1*H*)-ones (**13**) with a C3-C4 double bond included in Scifinder.

Once the diversity of the substituents in the various positions of the 1,6-naphthyridin-2(1*H*)-ones had been analyzed, a visual comparison of the diversities covered at positions C3, C4, C5, C7, C8, and N1 of structures **14** (C3-C4 single bond) and **13** (C3-C4 double bond) included in SciFinder is shown in Figure 3 and Figure 4.

As can be easily deduced, there is a lot of room to modify structures **14** and **13** because the degree of substitution is not very high and in nearly all positions the most present substituent is a hydrogen atom. Although the differences in the substitution pattern of **14** and **13** are certainly connected with the synthetic routes developed that favor one or another type of substituents, the different biological activities sought with each of the structures are undoubtedly the ultimate reason for such diversity.

## 3. Synthetic Approaches to 1,6-Naphthyridin-2(1*H*)-ones

**Figure 5:**
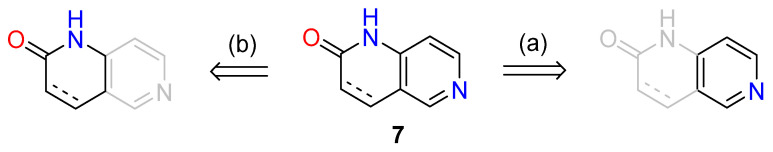
Synthetic approaches for 1,6-naphthyridin-2(1*H*)-ones (**7**): (**a**) from a preformed pyridine and (**b**) from a preformed pyridone.

From the diverse synthetic routes that can be envisaged for a bicyclic system such as the 1,6-naphthyridin-2(1*H*)-ones (**7**), in this review we have selected the two most common: (a) construction from a preformed pyridine and (b) construction from a preformed pyridone (Figure 5).

The retrieval of synthetic routes for a given structure is carried out in SciFinder by using two different protocols:

(1) *Reaction Structure* tool: it can be used in two ways, (a) drawing the substructures of two starting products and the reaction arrow without including the reaction product or (b) drawing the substructure of a possible starting material and the reaction arrow together with the structure of the reaction product. Both approaches are useful when the possible starting products are known.
(2) *Retrosynthetic* analysis tool: drawing the structure of the final product and indicating with a small arrow of the structure editor the bonds to be retrosynthetically broken. This approach is useful for exploring several possible disconnections from a given final structure.

In this review, it was necessary to use a combination of both protocols to draw a picture of the synthetic approaches used for the preparation of 1,6-naphthyridin-2(1*H*)-ones **14** and **13**.

### 3.1. Synthesis from a Preformed Pyridine

**Figure 6:**
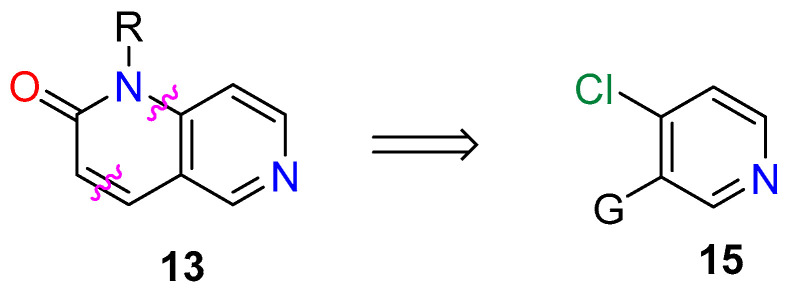
Synthetic approach for 1,6-naphthyridin-2(1*H*)-one (**13**) from a preformed 4-chloropyridine (**15**).

The searching methodologies described above allowed us to identify two synthetic approaches when a preformed pyridine is used as precursor:

(a) The use of an adequately substituted 4-chloropyridine (**15**) as the starting material, which corresponds to the disconnection of the N1-C8a and C3-C4 bonds of the 1,6-naphthyridin-2(1*H*)-one (**13**) bearing a C3-C4 double bond (Figure 6).

**Scheme 2:**
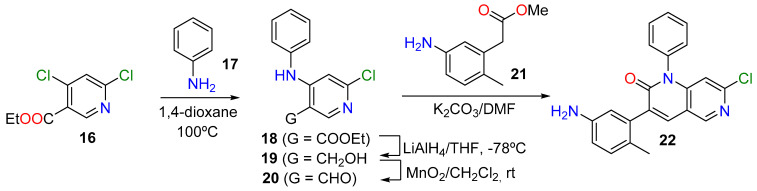
Synthesis of 1,6-naphthyridin-2(1*H*)-one (**22**) from 4,6-dichloro-3-pyridinecarboxylate (**16**).

There are in total seven references, all of them patents, that use such an approach. In five of them the chloro substituted pyridine is ethyl 4,6-dichloro-3-pyridinecarboxylate (**16**, corresponding to **15** with G = COOEt) [70,71,72,73,74], which is reacted with an amine, that introduces the N1 substituent of the final 1,6-naphthyridin-2(1*H*)-one, and is subsequently condensed with methyl phenylacetate to afford the corresponding structure **13**. An example of this approach is included in Scheme 2, in which **16** is treated with aniline (**17**) to yield the intermediate **18** (G = COOEt) that reduced to benzyl alcohol **19** (G = CH_2_OH) and subsequently oxidized to aldehyde **20** (G = CHO), which in turn is condensed with methyl phenylacetate **21** to yield 1,6-naphthyridin-2(1*H*)-one (**22**) in 40% of the global yield.

In the other two references, the chloro substituted pyridine bears an aldehyde (G = CHO) [75] or ketone group (G = COMe) [76]. It is noteworthy that there is not a single example of the construction of 1,6-naphthyridin-2(1*H*)-ones (**14**) with a C3-C4 single bond.

**Figure 7:**
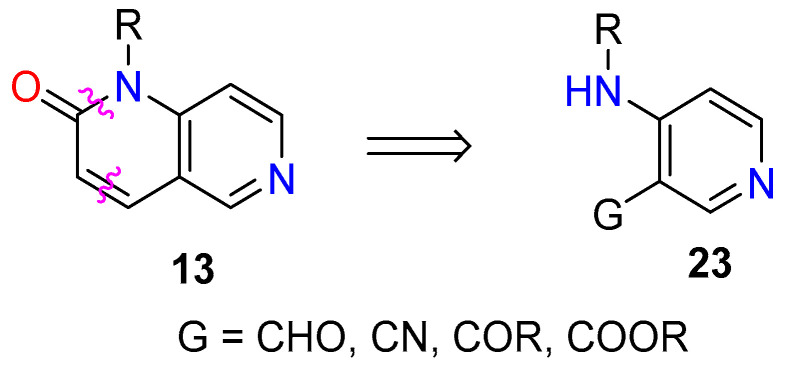
Synthetic approach for 1,6-naphthyridin-2(1*H*)-one (**13**) from a preformed 4-aminopyridine (**23**).

(b) The use as starting material of a preformed *N*-substituted or unsubstituted pyridine-4-amine (**23**) that includes a carbon functional group G (CHO, CN COOR, or COMe,) at position C3 of the pyridine ring. This approach corresponds to the disconnection of the N1–C2 and C3-C4 bonds of the 1,6-naphthyridin-2(1*H*)-one system (Figure 7). In the case of the CHO group, there are 76 references (50 of them patents) [77,78], 12 references for the CN group (4 patents) [79], and 5 references use a ketone group. This second approach is mainly used when the final 1,6-naphthyridin-2(1*H*)-one **13** is not bearing any substituent at N1 (R^1^ = H).

**Scheme 3:**
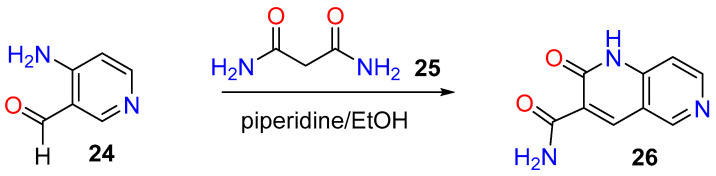
Synthesis of 1,6-naphthyridin-2(1*H*)-one (**26**) from 4-aminonicotinaldehyde (**24**).

An example of the use of a 4-aminonicotinaldehyde is the formation of **26** upon condensation of **24** with malonamide **25** to afford 1,6-naphthyridin-2(1*H*)-one (**26**) in the presence of piperidine and EtOH (Scheme 3). In this type of condensation, dimethyl malonate, methyl cyanocetate, or metyl phenylacetate can alternatively be used during the formation of the bicyclic system [78].

**Scheme 4:**
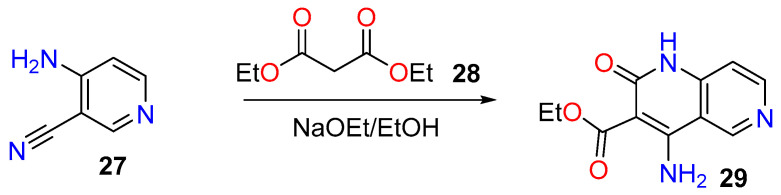
Synthesis of 1,6-naphthyridin-2(1*H*)-one (**29**) from 4-aminonicotinonitrile (**27**).

Scheme 4 shows the use of 4-aminonicotinonitrile (**27**) in the formation of the 1,6-naphthyridin-2(1*H*)-one. In this example, the condensation between **27** and diethyl malonate (**28**) in NaOEt in EtOH affords the corresponding bicyclic system **29**, bearing an amino group at position C4 [79].

**Scheme 5:**
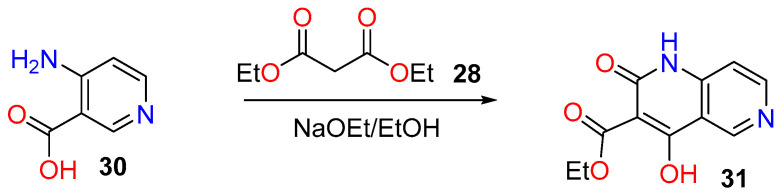
Synthesis of 1,6-naphthyridin-2(1*H*)-one (**31**) from 4-aminonicotinic acid (**30**).

Similarly, the use of 4-aminonicotinic acid (**30**), condensed with diethyl malonate (**28**), affords the corresponding 4-hydroxy substituted 1,6-naphthyridin-2(1*H*)-one **31** [47] (Scheme 5).

Finally, there is a single example of the use of this strategy for the synthesis of a 1,6-naphthyridin-2(1*H*)-one with a single C3-C4 bond [30].

### 3.2. Synthesis from a Preformed Pyridone

**Figure 8:**
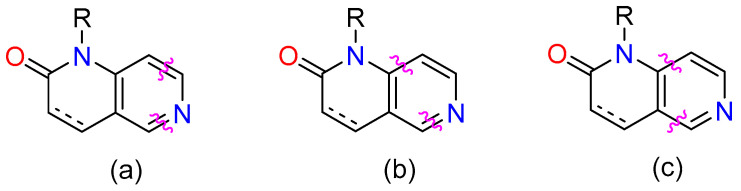
Synthetic approaches for 1,6-naphthyridin-2(1*H*)-ones (**7**) from a preformed pyridone.

In this second synthetic approach, we considered three possible disconnections of the 1,6-naphthyridin-2(1*H*)-ones (**7**): (a) in between C5–C6 and C7–C8, (b) in between C5–C6 and C8–C8a, and (c) in between C4a–C5 and C8–C8a (Figure 8). The disconnections were carried out using a C3-C4 undetermined bond to cover both levels of unsaturation.

**Scheme 6:**
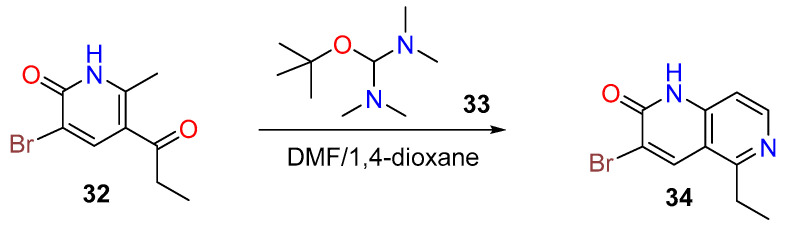
Synthesis of 1,6-naphthyridin-2(1*H*)-one (**34**) from pyridone (**32**).

In the case of disconnection (a), a total of 25 references were found, among which the works of Singh [80], Savarin [81], and Cywin [51] are especially representative. Thus, pyridone **32** treated with *tert*-butoxybis(dimethylamino)methane (**33**) affords the 1,6-naphthyridin-2(1*H*)-one (**34**) [80] (Scheme 6).

**Scheme 7:**
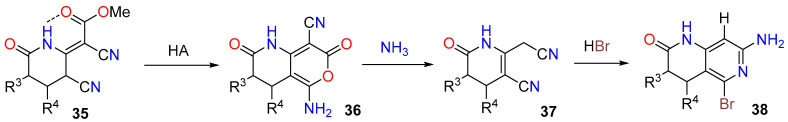
Synthesis of 8-unsubstituted 1,6-naphthyridin-2(1*H*)-ones (**38**) by cyclization of compounds **37** obtained by a ring opening, decarboxylation and loss of ammonia from pyrano[4,3-*b*]pyridine-2,7-diones (**36**).

Virtually the only methodology described that uses disconnection (b) is the one developed by our group in the early 1990s [8,9] (Scheme 1). The use of dimethyl malonate, metyl cyanoacetate, or cyanoacetamide, instead of malonitrile on pyridones **8** in combination with acidic or basic cyclizations on the corresponding intermediates referable to **10**, allowed the synthesis of a wide range of 1,6-naphthyridin-2(1*H*)-ones with a diverse set of substituents at positions C3, C4, C5, C7, and a nitrile group at C8, that present a C3-C4 single bond. When methyl cyanoacetate was used, the treatment of pyridones **8** with methyl cyanoacetate yielded the corresponding intermediates **35**, which present an intramolecular hydrogen bond between the COOMe group and the lactam NH group. The treatment of **35** in acidic media (HCl, HBr or TFA) afforded the corresponding pyrano[4,3-*b*]pyridine-2,7-dione (**36**) by cyclization of the ester group and the cyano group linked to the pyridone ring. Surprisingly, when compounds **36** were treated with ammonia, the cyanomethyl substituted pyridones **37** were formed (not accessible upon treatment of **8** with MeCN) through a ring opening, decarboxylation, and loss of ammonia [9]. Compounds **37** allowed the synthesis of C8-unsubstituted 1,6-naphthyridin-2(1*H*)-ones **38** upon treatment with HBr (Scheme 7).

**Scheme 8:**
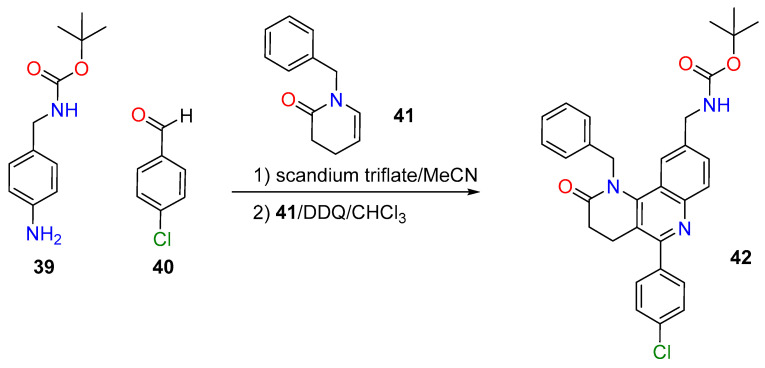
Synthesis of fused 1,6-naphthyridin-2(1*H*)-one (**42**) from pyridone (**41**).

As for the third disconnection, (c) is present in 17 references and a good example is the synthesis of the fused 1,6-naphthyridin-2(1*H*)-one **42** starting from **39** and **40** treated with pyridone **41** [82] (Scheme 8).

## 4. Biomedical Applications of 1,6-Naphthyridin-2(1*H*)-ones

SciFinder allows the retrieval of the biological activity of a single compound but makes it difficult to obtain the detailed biological activity for a large collection of compounds. The use of the filters *Biological Study* or *Therapeutic Use* on a group of structures allows the retrieval of all the references, including biological data, that later must be examined one by one. Nevertheless, using the *Concept* tool on the references retrieved under *Biological Study* in the case of the 1,6-naphthyridin-2(1*H*)-ones (**14**) with a C3-C4 single bond and on the 1,6-naphthyridin-2(1*H*)-ones (**13**) with a C3-C4 double bond (78 and 819 references, respectively), it is possible to obtain a list, ordered by frequency, of the indexed terms for the preferential biological targets of each structure (Table 4).

The results obtained clearly show that in the case of 1,6-naphthyridin-2(1*H*)-ones (**13**) with a C3-C4 double bond the most common use for this type of molecule is as an antitumor, as some of the most frequently indexed terms are: antitumor agent, neoplasm, melanoma, etc. In particular, 281 references included antitumor agents [49,83].

On the contrary, for 1,6-naphthyridin-2(1*H*)-ones (**14**) with a C3-C4 single bond the number of references found was lower. The most relevant biological activity of such molecules seems to be in the cardiovascular system. The most commonly indexed terms are: antihypertensives, cardiovascular agents, angiotensin II receptor antagonists, etc. Specifically, 16 references classify them as antihypertensives [84,85].

A search carried out in the Protein Data Bank (PDB, https://www.rcsb.org/, accessed on 27 September 2021) has shown the presence of five crystal structures in the case of 1,6-naphthyridin-2(1*H*)-ones (**14**) with a C3-C4 single bond and, apparently, none in the case of 1,6-naphthyridin-2(1*H*)-ones (**13**) with a C3-C4 double bond. The PDB entry 4YAY corresponds to the Human Angiotensin II Receptor complexed with the naphthyridine with CAS number 146709-78-6, while the 5VDH corresponds to the Human Glycine Receptor alpha-3 bound to the naphthyridine AM-3607 and Ivermectin.

An interesting aspect to know at this point in this review would be if any 1,6-naphthyridin-2(1*H*)-one-based product has reached the market. Such information is difficult to retrieve in SciFinder because this database does not clearly incorporate commercial information or the stages of development of a drug candidate. Therefore, we used Drugbank (https://www.drugbank.ca/, accessed on 17 July 2021), a database containing information on drugs and drug targets, for researching the market situation of compounds **13** and **14** [86].

**Figure 9:**
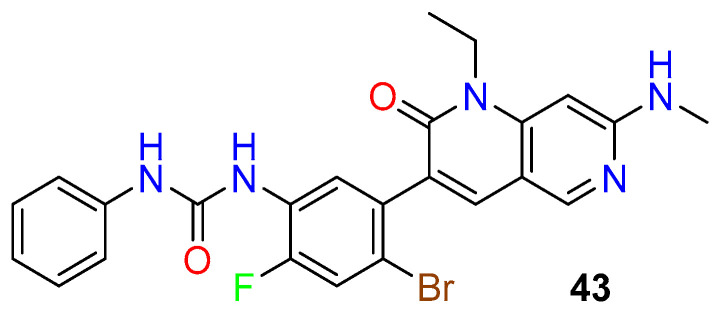
Ripretinib, also known as Qinlock, (**43**).

Our search showed that in the case of 1,6-naphthyridin-2(1*H*)-ones (**13**), a single compound bearing a C3-C4 double bond is included in the database. Ripretinib, also known as Qinlock, (**43**, Deciphera Pharmaceuticals, CAS 1442472-39-0, Figure 9), is a kinase inhibitor, approved by the FDA on 48 May 2020. It is used for the treatment of advanced gastrointestinal stromal tumors (GIST) that do not respond to other kinase inhibitors such as sunitinib and imatinib [87,88,89]. Ripretinib was approved as a fourth-line therapy after a pre-treatment with at least three or more kinase inhibitors.

## 5. Conclusions

In this paper, we reviewed the substitution patterns of 1,6-naphthyridin-2(1*H*)-ones (**13**) and (**14**) bearing a double and single C3-C4 bond, establishing the kind of substituents mainly used at positions N1, C3, C4, C5, C7, and C8 of such systems.

Two main synthetic strategies for the synthesis of such compounds were found: starting from a preformed pyridine or pyridone. The correlation between the structure of 1,6-naphthyridin-2(1*H*)-ones and their biological activity has been established, showing that the presence or absence of the C3-C4 double bond, together with the substitution pattern that our analysis has revealed, is responsible for the two different main biological activities of such compounds. Thus, we have shown that compounds **14**, with a C3-C4 single bond, have been mainly used in cardiovascular diseases, while compounds **13**, bearing a C3-C4 double bond, have been primarily used as antitumor agents. Repretinib, a 1,6-naphthyridin-2(1*H*)-one (**13**) bearing a C3-C4 double bond, has reached the market as a tyrosine–kinase inhibitor for the treatment of advanced gastrointestinal stromal tumors (GIST).

## Figures and Tables

**Table 1:** Substitution pattern at N1 of 1,6-naphthyridin-2(1*H*)-ones with a C5-C6 single bond (**14**) and with a C5-C6 double bond (**13**).

R ^1^	Structures 14 (%)	References ^1^	Structures 13 (%)	References
H	51.86	[12,13]	35.95	[14,15]
Me	1.65	[16,17]	10.47	[18,19]
Alkyl	5.77	[20,21]	7.29	[22,23]
Carbocycle	2.16	[20,24]	2.76	[25,26]
Ph	15.16	[24,27]	17.47	[28,29]
Heterocycle	4.12	[30,31]	4.29	[32,33]

^1^ The references included in Table 1, Table 2 and Table 3 have been selected using two criteria: (1) if possible, to select recent representative examples from the literature and (2), when available, selecting a paper published in a journal rather than a patent.

**Table 2:**
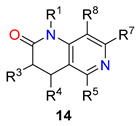
Substitution pattern at C5 and C7 of 1,6-naphthyridin-2(1*H*)-ones (**14**) with a C3-C4 single bond.

Substituent	R^5^		R^7^	
	Structures (%)	References	Structures (%)	References
H	78.19	49 [36,40]	65.87	42 [12,41]
C	1.05	56 [39,42]	25.65	72 [38,43]
N	0.77	9 [8,35]	2.28	12 [34,35]
O	5.27	9 [37,44]	5.62	7 [8,40]
X	14.69	11 [8,9]	0.47	11 [9,34]

**Table 3:**
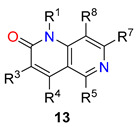
Substitution pattern at C5 and C7 of 1,6-naphthyridin-2(1*H*)-ones (**13**) with a C3-C4 double bond.

Substituent	R^5^		R^7^	
	Structures (%)	References	Structures (%)	References
H	66.59	808 [28,46]	33.98	246 [11,47]
C	20.69	113 [39,48]	43.25	614 [39,49]
N	3.92	19 [50,51]	16.34	125 [52,53]
O	8.25	41 [54,55]	4.20	29 [56,57]

**Table 4:** *Index terms* in SciFinder for compounds **14** and **13**.

Compounds 14		Compounds 13	
Index Term	Frequency	Index Term	Frequency
Angiotensin AT1 receptors	19	Antitumor agents	281
Cardiovascular agents	18	Signal transduction	235
Animals	17	Animals	205
Antihypertensives	16	Neoplasm	163
Angiotensin II receptor antagonists	15	Protein phosphorylation	135
Combination chemotherapy	13	Target of rapamycin complex 1	130
Heart failure	13	TOR Serine-Threonine Kinases	129
Antidiabetic agents	10	Autophagy	120
Antitumor agents	10	Cell proliferation	120
Antiarthritics	5	Lung neoplasm	55

## Data Availability

Data sharing not applicable.
